# Prize Essays

**Published:** 1873-10-01

**Authors:** 


PRIZE ESSAYS.—We hope, the members of the Association of American Medical Editors will not forget that there is a prize of $100 offered for the best essay on the question, "At what stage of pulmonary tuberculosis is a change of Climate desirable; what are the principles that should govern us in choosing the kind of change to be made. And which are the best localities in North America for patients of this class?"

The subject is a very important one. Competition is limited to members of the Association; and the essays must be sent to one of the members of the Committee on Prize Essays, on or before the first of April, 1874. The Committee are Drs. N. S. Davis, of Chicago, Illinois; J. P. Logan, of Atlanta, Georgia; and J. M. Toner, of Washington, District of Columbia. Will our exchanges call attention to this subject?

